# Favourable response of serum prostate-specific antigen to conjugated oestrogen in castrate-resistant prostate cancer in Jamaica

**DOI:** 10.3332/ecancer.2018.829

**Published:** 2018-04-24

**Authors:** Andrew Condappa, Maxine Gossell-Williams, William Aiken

**Affiliations:** 1 Department of Basic Medical Sciences, University of the West Indies, Kingston, Jamaica; 2 Department of Surgery, Anaesthesia and Intensive Care, University of the West Indies, Kingston, Jamaica

**Keywords:** androgen, castrate-resistant prostate cancer, conjugated oestrogen, premarin

## Abstract

Conjugated oestrogen is one of the more affordable secondary hormonal options available for castrate-resistant prostate cancer (CRPC) in Jamaica. The present study was conducted to examine the disease response in Jamaican men with CRPC treated with conjugated oestrogen. This study retrospectively reviewed the medical notes of patients who attended the urologic clinic of the University Hospital of the West Indies from 1 January 2009 to 31 December 2013 and a private urology clinic from 2 November 2005 to 3 June 2015 to identify patients diagnosed with CRPC treated with conjugated oestrogen (Premarin ®) as secondary therapy. The primary endpoint of favourable response, using the Prostate Cancer Clinical Trials Working Group 2 criteria is a decline of ≥50% in serum prostate-specific antigen (PSA) concentrations from baseline after treatment. The proportion of patients responding by the first 3-month follow-up visit and the maximal PSA declined over the 24 months of follow-up which were recorded. Thirty-two patients diagnosed with CRPC and treated with conjugated oestrogen were identified. All patients were prescribed 5.0 mg (2.5 mg tablets, twice daily) orally, as well as low dose aspirin. Favourable response was observed in 14 (43.8%) patients; however, eight other patients showed a decline in serum PSA concentration of <50%. There were no reported adverse effects. Conjugated oestrogen produced a PSA decline in Jamaican CRPC patients of this study and may therefore be a useful option for secondary therapy of CRPC. Further assessment is needed.

## Introduction

In Jamaica, prostate cancer is the most common cancer affecting men [1] and the leading cause of male cancer-related deaths [2]. Most prostate cancer cases are usually locally advanced or progress to metastatic disease [3] and are mainly treated by medical castration, the costs of which are subsidised by the National Health Fund. For patients who develop castrate resistant prostate cancer (CRPC), the secondary therapy from the National Health Fund subsidised list is the conjugated oestrogen preparation, Premarin®, commonly prescribed at a dose of 2.5 mg twice daily. However, oestrogen (conjugated or as diethylstilbestrol) is not the preferred therapy for CRPC, as it is not as selective as newer alternatives and doses higher than 1 mg per day are associated with increased risk of cardiovascular complications [4–6]. With oestrogen remaining a popular choice for CRPC therapy in Jamaica, an assessment of clinical benefit is important in this group of patients.

In the absence of other clinical parameters of improved outcome in patients with prostate cancer, the Prostate Cancer Clinical Trials Working Group 2 determined that the percentage decline in prostate-specific antigen (PSA) from baseline to 12 weeks (or earlier for those who discontinue therapy), as well as the maximum decline in PSA that occurs at any point after treatment, be reported as a surrogate marker for favourable response [7]. In addition, clinical data suggests that PSA decline of at least 50% indicates a higher survival advantage for patients [8]. The aim of this study is to examine the disease response in Jamaican men with CRPC treated with conjugated oestrogen.

## Materials and methods

The study was reviewed and approved by the Ethics Committee of the University of the West Indies. A retrospective review of the medical notes of patients who attended the urology clinic at the University Hospital of the West Indies (1 January 2009–31 December 2013) and patients from a private urology clinic (2 November 2005–3 June 2015) was conducted. Patients attending these clinics are booked for follow-up visits every three months. Patients diagnosed with CRPC and placed on conjugated oestrogen, 2.5 mg twice daily with low dose aspirin were included in this study. The variables recorded from the medical records included the information on initial androgen depravation therapy prescribed for hormone-naïve prostate cancer, oestrogen dosage, the PSA readings recorded up to 24 months or until oestrogen therapy was terminated. Pathological details on the presence and location of metastases were recorded as documented by physicians’ notes; any documented adverse events were also recorded.

Patient characteristics were described using median and interquartile range (IQR) for continuous variables; frequency and proportion were used for categorical variables. The statistical analysis was done using Statistical Package for Social Sciences, version 20. Waterfall charts were used to graphically display the PSA response as percentage change in PSA at first 3- month visit and maximal change (which is at any point in time on therapy on oestrogen within the 24 months). Assessment of the PSA response to therapy was based on guidelines from Prostate Cancer Clinical Trials Working Group 2 with PSA decline of ≥50% defined as a favourable response.

## Results

Thirty-two patients were identified with CRPC. Table 1 provides the baseline characteristic of the subjects.

**Figure 1.**
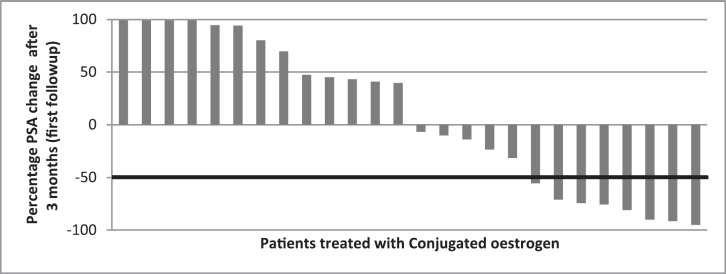
Waterfall chart showing first follow-up PSA response in patients treated with conjugated oestrogen. The horizontal line at −50% represents marker of favourable response. At first 3-month visit, eight subjects showed ≥50% PSA decline.

**Figure 2.**
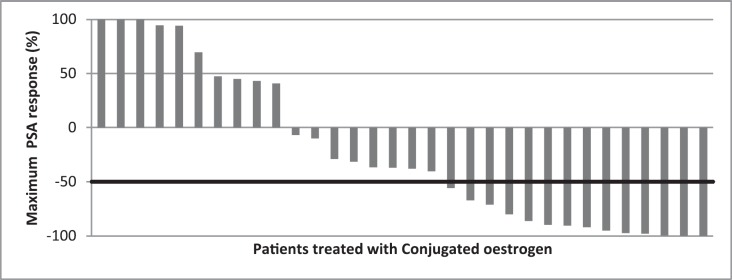
Waterfall chart showing maximum PSA response in patients treated with conjugated oestrogen. The horizontal line at −50% represents marker of favourable response. Favourable response (≥ 50% PSA decline) was seen in 14 subjects, while eight others showed led than 50% PSA decline.

The baseline PSA of the subjects ranged from 2.10 to 2000 ng/mL; PSA changes at first 3-month visit and maximum percentage PSA change were examined and reported using waterfall charts. At the first 3-month visit, PSA test results were recorded for 26 of the 32 patients; results were missing for six subjects. Eight patients (30.8%) obtaining favourable PSA response, while thirteen (50%) subjects had increase in PSA (Figure 1). Maximum PSA change observed over the 24 months for all patients showed that of the total 32 patients treated with conjugated oestrogen, 14 (43.8%) had favourable PSA decline (Figure 2).

No adverse drug reaction associated with oestrogen therapy was reported nor was oestrogen therapy terminated due to adverse effect.

## Discussion

With oestrogen being the popular option for secondary therapy of CRPC, evaluation of improved clinical outcomes is important. CRPC patients identified in this study were all prescribed conjugated oestrogen at a dose of 2.5 mg twice daily (5.0 mg). Approximately 44% of the patients treated, had a favourable PSA decline during the 24 months. A review of published studies among men with CRPC found variability in a favourable PSA response in patients receiving oestrogens ranging from 25% to 71%, with all the studies reporting oral doses from 1 to 4 mg [9–17]. Our response rate is thus comparable to other studies and therefore suggests that oestrogen may constitute a valuable therapy among Jamaican patients. The assessment of PSA decline is, however, only a biochemical marker of outcomes and overall assessment of clinical improvement requires follow up on changes in bone, symptoms and soft-tissue [1].

The amounts of oestrogen prescribed to patients were at higher doses than identified in published studies of similar patients. This is the standard dose prescribed by urologists in our setting and low dose aspirin, which is also part of standard therapy, is expected to reduce the risk of thrombotic events. Among this group, there was no documentation of adverse events or complications requiring oestrogen withdrawal.

Another parameter used to determine the benefit of drug therapy for CRPC established by the Prostate Cancer Clinical Trials Working Group 2 is time to disease progression; which is a measurement related to consecutive elevated readings of PSA from baseline [7]. Although patients are scheduled for follow-up visits every three months, the patient’s medical notes reflected absence of regular PSA data and therefore calculation of this parameter was one limitation of this study. Other limitations relate to using biochemical data that were obtained from different laboratories, as well as lack of information to facilitate assessment of adherence to therapy.

## Conclusion

Conjugated oestrogen provided favourable response in Jamaican CRPC patients with maximal PSA decline of ≥50% in approximately 44% of patients and therefore may be considered as having some benefit on this biochemical marker of the disease. With oestrogen remaining on the subsidised list of the National Health Fund, further assessment of the therapeutic benefit is needed.

## Conflicts of interest

None of the authors have any conflict of interest to report pertinent to the work in this paper.

## Figures and Tables

**Table 1.** Baseline characteristics at initiation of conjugated oestrogen in CRPC patients.

Patient characteristics	Conjugated oestrogen (*n* = 32)
**Age at start of therapy in years, *median (IQR)***	80(12)
**Baseline PSA in ng/mL, *median (IQR)***	45(129.55)
**Baseline testosterone levels in ng/dL, *median (IQR)***^a^	0.32(0.46)
**Biopsy Gleason score, *n (%)***	
**≤6**	1(3.1%)
**=7**	7(21.9%)
**≥8**	15(46.9%)
**Unknown**	9(28.1%)
**Metastasis sites, *n* (%)**	
**Bone**	8(25%)
**Multiple swites**	1(3.1%)
**Unknown**	23(71.9 %)
**Androgen depravation therapy used before conjugated oestrogen, *n* (%)**	
**Zoladex (goserelin)**	8 (25%)
**Casodex (bicalutamide)**	3 (9.4%)
**Androcour (cyproterone acetate)**	5 (15.6%)
**Casodex + Zoladex**	7 (21.9%)
**Flutamide + Zoladex**	1 (3.1%)
**Unknown**	8 (25%)

a Median baseline testosterone levels for 8/32 patients; testosterone levels for 24/32 patients were not available during review
